# Supplementary material for: Potential of Trichoderma virens HZA14 in Controlling Verticillium Wilt Disease of Eggplant and Analysis of Its Genes Responsible for Microsclerotial Degradation
Source: Plants (Basel). 2023 Nov 3;12(21):3761. doi: 10.3390/plants12213761 (PMC10649075; doi:10.3390/plants12213761)
Supplement: Supplementary file 1 [file plants-12-03761-s001.zip › plants-2564833-supplementary.pdf]

Table S1. The summary of filtrating of data from 5 samples

Sample	Low-quality Reads Number	Low-quality Reads Rate (%)	Ns Reads Number	Ns Reads Rate (%)	Adapter Polluted Reads Number	Adapter Polluted Reads Rate (%)	Raw Q30 Bases Rate (%)	Clean Q30 Bases Rate (%)
3d1	247,666	0.51	80,350	0.17	1,422,250	2.92	94.28	94.53
3d2	285,374	0.62	119,458	0.26	1,682,376	3.65	94.03	94.36
3d3	258,734	0.56	104,576	0.23	1,955,898	4.21	94.7	95
6d1	167,088	0.36	107,152	0.23	1,228,146	2.65	92.78	92.99
6d2	168,518	0.35	99,602	0.21	1,378,994	2.86	92.36	92.55
6d3	218,044	0.51	74,086	0.17	372,114	0.87	91.48	91.7
9d1	272,842	0.59	113,518	0.24	774,844	1.68	92.17	92.46
9d2	304,430	0.72	115,352	0.27	578,148	1.38	92.22	92.56
9d3	373,630	0.86	72,444	0.17	1,463,248	3.39	91.34	91.8
12d1	294,770	0.62	137,014	0.29	577,152	1.22	91.66	91.95
12d2	231,074	0.51	117,782	0.26	617,152	1.37	92.32	92.58
12d3	287,424	0.63	277,632	0.61	667,408	1.46	92.83	93.23
15d1	223,532	0.47	41,360	0.09	1,052,474	2.23	94.46	94.67
15d2	250,698	0.5	90,734	0.18	1,994,356	3.96	94.05	94.31
15d3	217,032	0.44	38,672	0.08	1,686,662	3.43	94.47	94.67
Total	3,800,856	8	1,589,732	3	17,451,222	37	1,395	1,399
Average	253,390	1	105,982	0	1,163,415	2	93.01	93.29067

Table S2. The summary of RNA-seq data from 5 samples.

Treatments	Rep.	Raw Reads Number	Clean Reads		Mapped reads	
			Total	Raw reads	Total	Clean reads
3th day	1	48,694,098	46,943,832	96.41	45,610,346	97.16
	2	46,132,964	44,045,756	95.48	42,853,129	97.29
	3	46,500,864	44,181,656	95.01	43,061,345	97.46
6th day	1	46,396,834	44,894,448	96.76	26,230,319	58.43
	2	48,263,138	46,616,024	96.59	38,260,921	82.08
	3	42,955,580	42,291,336	98.45	30,140,380	71.27
9th day	1	46,271,222	45,110,018	97.49	25,308,591	56.10
	2	41,973,028	40,975,098	97.62	19,843,293	48.43
	3	43,182,142	41,272,820	95.58	17,491,240	42.38
12th day	1	47,160,676	46,151,740	97.86	41,313,003	89.52
	2	45,191,892	44,225,884	97.86	33,912,174	76.68
	3	45,729,560	44,497,096	97.31	40,747,833	91.57
15th day	1	47,125,420	45,808,054	97.20	44,612,998	97.39
	2	50,318,454	47,982,666	95.36	46,082,876	96.04
	3	49,164,158	47,221,792	96.05	45,765,333	96.92
Total	15	695,060,030	672,218,220	1,451	541,233,781	1198.72
Average	3	46,337,335	44,814,548	97	36,082,252	79.91466667

Figure S1. Visualization of the DEGs by volcano plot.

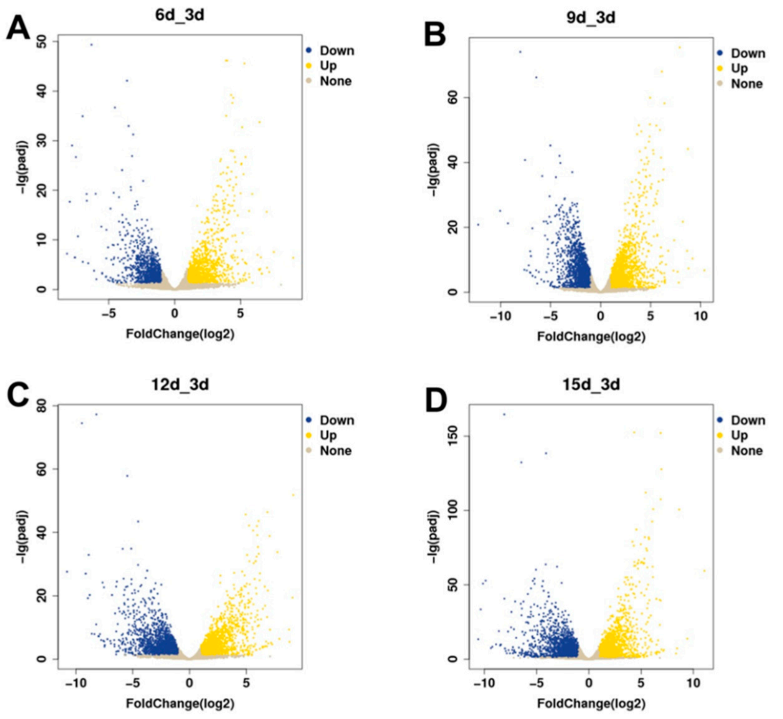

Table S3. Gene ontology classification of DEGs

6d-3d					
**Ontology**	**Term_name**	**Up_ Count**	**Up_ Percent**	**Down_ Count**	**Down_ Percent**
cellular_component	extracellular region	34	2.84	62	5.21
cellular_component	cell	3	0.25	2	0.17
cellular_component	nucleoid	1	0.08	2	0.17
cellular_component	membrane	132	11.03	107	8.98
cellular_component	cell junction	1	0.08	6	0.5
cellular_component	membrane-enclosed lumen	8	0.67	17	1.43
cellular_component	protein-containing complex	55	4.59	103	8.65
cellular_component	organelle	233	19.47	238	19.98
cellular_component	other organism part	1	0.08	2	0.17
cellular_component	extracellular region part	7	0.58	15	1.26
cellular_component	organelle part	137	11.45	160	13.43
cellular_component	membrane part	172	14.37	163	13.69
cellular_component	synapse part	3	0.25	5	0.42
cellular_component	cell part	384	32.08	452	37.95
cellular_component	synapse	0	0	2	0.17
cellular_component	supramolecular complex	2	0.17	13	1.09
Total		1173	97.99	1349	113.27
biological_process	reproduction	9	0.75	0	0

biological_process	cell killing	2	0.17	0	0
biological_process	immune system process	2	0.17	4	0.34
biological_process	behavior	1	0.08	2	0.17
biological_process	metabolic process	280	23.39	439	36.86
biological_process	cell proliferation	1	0.08	0	0
biological_process	carbohydrate utilization	0	0	1	0.08
biological_process	cellular process	334	27.9	459	38.54
biological_process	nitrogen utilization	1	0.08	0	0
biological_process	reproductive process	24	2.01	11	0.92
biological_process	biological adhesion	1	0.08	0	0
biological_process	signaling	3	0.25	2	0.17
biological_process	multicellular organismal process	13	1.09	12	1.01
biological_process	developmental process	32	2.67	25	2.1
biological_process	growth	12	1	3	0.25
biological_process	locomotion	3	0.25	4	0.34
biological_process	rhythmic process	1	0.08	2	0.17
biological_process	response to stimulus	67	5.6	46	3.86
biological_process	localization	104	8.69	109	9.15
biological_process	multi-organism process	30	2.51	38	3.19
biological_process	biological regulation	128	10.69	90	7.56
biological_process	cellular component organization or biogenesis	73	6.1	82	6.88
biological_process	cell aggregation	2	0.17	1	0.08
biological_process	detoxification	4	0.33	10	0.84
Total		1127	94.14	1340	112.51
molecular_function	catalytic activity	368	30.74	560	47.02
molecular_function	structural molecule activity	7	0.58	67	5.63
molecular_function	transporter activity	74	6.18	68	5.71
molecular_function	binding	331	27.65	384	32.24
molecular_function	antioxidant activity	4	0.33	7	0.59
molecular_function	translation regulator activity	0	0	1	0.08
molecular_function	nutrient reservoir activity	1	0.08	1	0.08
molecular_function	molecular transducer activity	5	0.42	1	0.08
molecular_function	toxin activity	2	0.17	2	0.17
molecular_function	molecular function regulator	15	1.25	13	1.09
molecular_function	molecular carrier activity	0	0	1	0.08
molecular_function	transcription regulator activity	44	3.68	15	1.26
Total		851	71.08	1120	94.03
**9d-3d**					
**Ontology**	**Term_name**	**Up_ Count**	**Up_ Percent**	**Down_ Count**	**Down_ Percent**
cellular_componen	extracellular region	56	3.19	127	6.47
cellular_componen	cell	4	0.23	3	0.15
cellular_componen	nucleoid	1	0.06	4	0.2

cellular_componen	membrane	184	10.47	199	10.14
cellular_componen	cell junction	7	0.4	8	0.41
cellular_componen	membrane-enclosed lumen	7	0.4	42	2.14
cellular_componen	protein-containing complex	119	6.77	221	11.26
cellular_componen	organelle	394	22.41	453	23.08
cellular_componen	other organism part	1	0.06	2	0.1
cellular_componen	extracellular region part	13	0.74	27	1.38
cellular_componen	organelle part	232	13.2	306	15.59
cellular_componen	membrane part	237	13.48	283	14.42
cellular_componen	synapse part	8	0.46	6	0.31
cellular_componen	cell part	611	34.76	836	42.59
cellular_componen	synapse	5	0.28	4	0.2
cellular_componen	supramolecular complex	3	0.17	20	1.02
Total		1882	107.08	2541	129.46
biological_process	reproduction	12	0.68	4	0.2
biological_process	cell killing	3	0.17	0	0
biological_process	immune system process	3	0.17	9	0.46
biological_process	behavior	4	0.23	1	0.05
biological_process	metabolic process	449	25.54	738	37.6
biological_process	cell proliferation	2	0.11	0	0
biological_process	carbohydrate utilization	0	0	1	0.05
biological_process	cellular process	534	30.38	780	39.74
biological_process	nitrogen utilization	2	0.11	0	0
biological_process	reproductive process	43	2.45	24	1.22
biological_process	biological adhesion	5	0.28	0	0
biological_process	signaling	3	0.17	2	0.1
biological_process	multicellular organismal process	18	1.02	16	0.82
biological_process	developmental process	55	3.13	39	1.99
biological_process	growth	18	1.02	6	0.31
biological_process	locomotion	4	0.23	5	0.25
biological_process	rhythmic process	1	0.06	1	0.05
biological_process	response to stimulus	108	6.14	88	4.48
biological_process	localization	157	8.93	193	9.83
biological_process	multi-organism process	47	2.67	62	3.16
biological_process	biological regulation	239	13.59	168	8.56
biological_process	cellular component organization or biogenesis	126	7.17	143	7.28
biological_process	cell aggregation	3	0.17	1	0.05
biological_process	detoxification	6	0.34	19	0.97
Total		1842	104.76	2300	117.17
molecular_function	catalytic activity	502	28.56	918	46.77
molecular_function	structural molecule activity	9	0.51	90	4.58
molecular_function	transporter activity	98	5.57	122	6.21

molecular_function	binding	504	28.67	664	33.83
molecular_function	antioxidant activity	8	0.46	13	0.66
molecular_function	protein tag	0	0	3	0.15
molecular_function	translation regulator activity	0	0	1	0.05
molecular_function	nutrient reservoir activity	0	0	3	0.15
molecular_function	molecular transducer activity	6	0.34	4	0.2
molecular_function	toxin activity	3	0.17	4	0.2
molecular_function	molecular function regulator	25	1.42	23	1.17
molecular_function	molecular carrier activity	1	0.06	1	0.05
molecular_function	transcription regulator activity	90	5.12	22	1.12
Total		1246	70.88	1868	95.14
12d-3d					
**Ontology**	**Term_name**	**Up_ Count**	**Up_ Percent**	**Down_ Count**	**Down_ Percent**
cellular_component	extracellular region	43	2.22	108	5.27
cellular_component	cell	5	0.26	4	0.2
cellular_component	nucleoid	0	0	6	0.29
cellular_component	membrane	201	10.38	208	10.15
cellular_component	cell junction	5	0.26	9	0.44
cellular_component	membrane-enclosed lumen	12	0.62	45	2.2
cellular_component	protein-containing complex	225	11.62	213	10.39
cellular_component	organelle	549	28.36	471	22.98
cellular_component	other organism part	1	0.05	2	0.1
cellular_component	extracellular region part	11	0.57	22	1.07
cellular_component	organelle part	351	18.13	321	15.66
cellular_component	membrane part	249	12.86	285	13.9
cellular_component	synapse part	5	0.26	7	0.34
cellular_component	cell part	807	41.68	845	41.22
cellular_component	synapse	3	0.15	5	0.24
cellular_component	supramolecular complex	5	0.26	21	1.02
Total		2472	127.68	2572	125.47
biological_process	reproduction	10	0.52	3	0.15
biological_process	cell killing	2	0.1	0	0
biological_process	immune system process	6	0.31	9	0.44
biological_process	behavior	3	0.15	2	0.1
biological_process	metabolic process	555	28.67	748	36.49
biological_process	cell proliferation	6	0.31	0	0
biological_process	carbohydrate utilization	0	0	1	0.05
biological_process	cellular process	681	35.18	786	38.34
biological_process	nitrogen utilization	3	0.15	0	0
biological_process	reproductive process	55	2.84	29	1.41
biological_process	biological adhesion	5	0.26	2	0.1
biological_process	signaling	8	0.41	1	0.05

biological_process	multicellular organismal process	20	1.03	17	0.83
biological_process	developmental process	62	3.2	34	1.66
biological_process	growth	23	1.19	9	0.44
biological_process	locomotion	4	0.21	4	0.2
biological_process	rhythmic process	1	0.05	2	0.1
biological_process	response to stimulus	145	7.49	95	4.63
biological_process	localization	176	9.09	208	10.15
biological_process	multi-organism process	53	2.74	61	2.98
biological_process	biological regulation	337	17.41	185	9.02
biological_process	cellular component	181	9.35	156	7.61
biological_process	cell aggregation	3	0.15	1	0.05
biological_process	detoxification	5	0.26	19	0.93
Total		2344	121.07	2372	115.73
molecular_function	catalytic activity	527	27.22	923	45.02
molecular_function	structural molecule activity	10	0.52	87	4.24
molecular_function	transporter activity	98	5.06	126	6.15
molecular_function	binding	591	30.53	660	32.2
molecular_function	antioxidant activity	8	0.41	18	0.88
molecular_function	protein tag	0	0	4	0.2
molecular_function	translation regulator activity	1	0.05	1	0.05
molecular_function	nutrient reservoir activity	2	0.1	1	0.05
molecular_function	molecular transducer activity	8	0.41	3	0.15
molecular_function	toxin activity	2	0.1	2	0.1
molecular_function	molecular function regulator	46	2.38	23	1.12
molecular_function	molecular carrier activity	1	0.05	1	0.05
molecular_function	transcription regulator activity	127	6.56	25	1.22
Total		1421	73.39	1874	91.43
15d-3d					
**Ontology**	**Term_name**	**Up_ Count**	**Up_ Percent**	**Down_ Count**	**Down_ Percent**
cellular_component	extracellular region	41	2.14	147	6.95
cellular_component	cell	2	0.1	3	0.14
cellular_component	nucleoid	0	0	2	0.09
cellular_component	membrane	189	9.87	214	10.12
cellular_component	cell junction	2	0.1	9	0.43
cellular_component	membrane-enclosed lumen	11	0.57	32	1.51
cellular_component	protein-containing complex	178	9.3	176	8.33
cellular_component	organelle	471	24.61	460	21.76
cellular_component	other organism part	1	0.05	1	0.05
cellular_component	extracellular region part	5	0.26	26	1.23
cellular_component	organelle part	289	15.1	292	13.81
cellular_component	membrane part	236	12.33	274	12.96
cellular_component	synapse part	4	0.21	9	0.43

cellular_component	cell part	706	36.89	814	38.51
cellular_component	synapse	3	0.16	3	0.14
cellular_component	supramolecular complex	3	0.16	19	0.9
Total		2141	111.85	2481	117.36
biological_process	reproduction	10	0.52	6	0.28
biological_process	cell killing	2	0.1	0	0
biological_process	immune system process	5	0.26	7	0.33
biological_process	behavior	2	0.1	2	0.09
biological_process	metabolic process	499	26.07	708	33.49
biological_process	cell proliferation	2	0.1	1	0.05
biological_process	carbohydrate utilization	0	0	1	0.05
biological_process	cellular process	602	31.45	766	36.23
biological_process	nitrogen utilization	4	0.21	0	0
biological_process	reproductive process	48	2.51	31	1.47
biological_process	biological adhesion	3	0.16	0	0
biological_process	signaling	6	0.31	3	0.14
biological_process	multicellular organismal process	15	0.78	16	0.76
biological_process	developmental process	54	2.82	49	2.32
biological_process	growth	20	1.04	10	0.47
biological_process	locomotion	5	0.26	4	0.19
biological_process	rhythmic process	0	0	3	0.14
biological_process	response to stimulus	139	7.26	109	5.16
biological_process	localization	165	8.62	183	8.66
biological_process	multi-organism process	52	2.72	67	3.17
biological_process	biological regulation	282	14.73	195	9.22
biological_process	cellular component	141	7.37	162	7.66
biological_process	cell aggregation	5	0.26	1	0.05
biological_process	detoxification	7	0.37	20	0.95
Total		2068	108.02	2344	110.88
molecular_function	catalytic activity	524	27.38	923	43.66
molecular_function	structural molecule activity	9	0.47	85	4.02
molecular_function	transporter activity	102	5.33	103	4.87
molecular_function	binding	566	29.57	653	30.89
molecular_function	antioxidant activity	9	0.47	23	1.09
molecular_function	protein tag	0	0	4	0.19
molecular_function	translation regulator activity	1	0.05	0	0
molecular_function	nutrient reservoir activity	2	0.1	2	0.09
molecular_function	molecular transducer activity	10	0.52	4	0.19
molecular_function	toxin activity	3	0.16	3	0.14
molecular_function	molecular function regulator	28	1.46	25	1.18
molecular_function	molecular carrier activity	1	0.05	2	0.09
molecular_function	transcription regulator activity	120	6.27	27	1.28
Total		1375	71.83	1854	87.69

Tables S4. Reverse transcription of total RNA to cDNA and genomic DNA removal reaction.

Reagent	Usage amount/μl
5 × gDNA Eraser Buffer	2.0
gDNA Eraser	1.0
Total RNA	20 reaction system <1 μg
RNase Free H_2O	10

The reagent-added samples were reacted at 42°C for 2 min and stored at 4°C.

Tables S5. Reverse transcription reaction when master mix prepare according to the amount of the reaction number +1.

Reagent	Usage amount/μl
5 × PrimerScript Buffer 2 (for Real Time)	4.0
PrimerScript RT Enzyme Mix I	1.0
RT Primer Mix	1.0
Step (1) reaction solution	10.0
RNase Free H_2O	2.0

The reagent-added sample was reacted at 37°C for 15 min, then reacted at 85°C for 5 sec, and finally stored at -20°C.

Tables S6. Fluorescence quantification to reaction system with Takara kit, instrument Bio-Rad CFX96.

Reagent	Usage amount/μl
PCR F-Primer（10 μmol/l）	0.4
PCR R-Primer（10 μmol/l）	0.4
cDNA	1.0
TB Green *Premix Ex Taq* II	10.0
RNase Free H_2O	8.2
